# Draft Genome Sequence of the Polychlorinated Biphenyl-Degrading Bacterium Cupriavidus basilensis KF708 (NBRC 110671) Isolated from Biphenyl-Contaminated Soil

**DOI:** 10.1128/genomeA.00143-15

**Published:** 2015-03-19

**Authors:** Hikaru Suenaga, Atsushi Yamazoe, Akira Hosoyama, Nobutada Kimura, Jun Hirose, Takahito Watanabe, Hidehiko Fujihara, Taiki Futagami, Masatoshi Goto, Kensuke Furukawa

**Affiliations:** a Bioproduction Research Institute, National Institute of Advanced Industrial Science and Technology (AIST), Tsukuba, Japan; b Biological Resource Center, National Institute of Technology and Evaluation (NITE), Tokyo, Japan; c Faculty of Engineering, Department of Applied Chemistry, University of Miyazaki, Miyazaki, Japan; d Research Institute for Sustainable Humanosphere, Kyoto University, Kyoto, Japan; e Department of Food and Nutrition, Beppu University, Beppu, Japan; f Faculty of Agriculture, Education and Research Center for Fermentation Studies, Kagoshima University, Kagoshima, Japan; g Faculty of Agriculture, Laboratory of Future Creation Microbiology, Kyushu University, Fukuoka, Japan

## Abstract

We report the draft genome sequence of Cupriavidus basilensis KF708 (NBRC 110671), which utilizes biphenyl as a sole carbon source and degrades polychlorinated biphenyls (PCBs). The KF708 strain possesses genes for biphenyl catabolism and other genes involved in various aromatic compounds.

## GENOME ANNOUNCEMENT

Due to their chemical and physical stabilities, polychlorinated biphenyls (PCBs) have been widely used for a variety of industrial purposes, and in the process have become serious environmental contaminants at a global level. Biphenyl-utilizing bacteria cometabolize PCBs into chlorobenzoic acids using biphenyl-catabolic enzymes. Since then, we have isolated 14 PCB-degrading bacterial strains (KF strains), including Cupriavidus basilensis KF708 (formerly known as Alcaligenes sp. strain KF708), from the soil near a biphenyl manufacturing plant in Kitakyushu, Japan by enrichment culture with biphenyl as a sole carbon source (1). These KF strains belong to phylogenetically distinct genera and exhibit specific growth characteristics on various biphenyl derivatives (1). The *bph* gene cluster involved in biphenyl/PCB degradation was cloned from one of these strains, Pseudomonas pseudoalcaligenes KF707, for the first time (2, 3). These KF strains, therefore, are a suitable model for investigating diversity, distribution, and evolution of *bph* genes and PCB-degrading bacteria in the biphenyl-contaminated soil. Here, we present the genomic features of the KF708 strain.

The draft genome sequence was determined by the National Institute of Technology and Evaluation (NITE) using a combined strategy of 454 GS FLX+ (Roche), MiSeq (Illumina), and HiSeq 1000 (Illumina) technologies. A standard fragment library was constructed for 454 sequencing, and 93,079 reads (62,943,993 bases) were obtained, while the pair-end sequencing with Illumina generated 5,948,932 reads (556,752,511 bases). The obtained reads were assembled using the Newbler software package (v2.6; Roche). The assembled genome is composed of 62 contigs (>500 bp) totaling 7,826,077 bases, with a G+C content of 68.8%. The *N*_50_ contig size and the largest contig size were 315,839 bp and 823,591 bp, respectively.

The draft genome sequence of the KF708 strain was uploaded to the RAST (Rapid Annotation using Subsystem Technology) server (http://rast.nmpdr.org) (4). The result described 7,104 predicted coding DNA sequences (CDSs), three rRNAs (one each of 5S, 16S, and 23S), and 59 tRNA sequences. This RAST-based annotation revealed the presence of 526 subsystems. A large number of genes (*n* = 207) involved in the metabolism of aromatic compounds were detected. These consisted of CDSs involved in biphenyl degradation (*n* = 19), the catechol *ortho*-cleavage pathway (*n* = 18), salicylate and gentisate catabolism (*n* = 13), protocatechuate catabolism (*n* = 22), the 4-hydroxyphenylacetic acid catabolic pathway (*n* = 21), and the homogentisate pathway of aromatic compound degradation (*n* = 40). The *bph* gene cluster (*bphEGFA1A2A3BCDA4*) was found in a single contig and was different from that of the KF707 strain and similar to that of Acidovorax sp. strain KKS102 (5) in terms of gene organization and the amino acid sequence of the corresponding enzymes.

### Nucleotide sequence accession numbers.

The draft genome sequence has been deposited at DDBJ/EMBL/GenBank under the accession numbers BBQM01000001 to BBQM01000062.
